# Maximizing Muscle Function in Cuff-Deficient Shoulders: A Rehabilitation Proposal for Reverse Arthroplasty

**DOI:** 10.1177/24715492211023302

**Published:** 2021-07-01

**Authors:** Helen Razmjou, Varda van Osnabrugge, Mark Anunciacion, Andrea Nunn, Darren Drosdowech, Ania Roszkowski, Analia Szafirowicz, Dragana Boljanovic, Amy Wainwright, Diane Nam

**Affiliations:** 1 Holland Bone and Joint Program, Holland Orthoapaedic & Arthritic Centre, Sunnybrook Health Sciences Centre, Toronto, Ontario, Canada; 2 Department of Physical Therapy, Faculty of Medicine, University of Toronto, Toronto, Ontario, Canada; 3 Sunnybrook Research Institute, Sunnybrook Health Sciences Centre, Toronto, Ontario, Canada; 4 Roth|McFarlane Hand & Upper Limb Centre, St. Joseph’s Health Care, London, Ontario, Canada; 5 Division of Orthopaedic Surgery, Department of Surgery, Faculty of Medicine, Western University, London, Ontario, Canada; 6 Division of Orthopedic Surgery, Department of Surgery, Sunnybrook Health Sciences Centre, Toronto, Ontario, Canada; 7 Division of Orthopaedic Surgery, Department of Surgery, Faculty of Medicine, University of Toronto, Toronto, Ontario, Canada

**Keywords:** biomechanics, complications, cuff tear arthropathy, deltoid, rehabilitation

## Abstract

**Purpose:**

The purpose of this review is to describe the role of altered joint biomechanics in reverse shoulder arthroplasty and to propose a rehabilitation protocol for a cuff-deficient glenohumeral joint based on the current evidence.

**Methods and Materials:** The proposed rehabilitation incorporates the principles of pertinent muscle loading while considering risk factors and surgical complications.

**Results:**

In light of altered function of shoulder muscles in reverse arthroplasty, scapular plane abduction should be more often utilized as it better activates deltoid, teres minor, upper trapezius, and serratus anterior. Given the absence of supraspinatus and infraspinatus and reduction of external rotation moment arm of the deltoid in reverse arthroplasty, significant recovery of external rotation may not occur, although an intact teres minor may assist external rotation in the elevated position.

**Conclusion:**

Improving the efficiency of deltoid function before and after reverse shoulder arthroplasty is a key factor in the rehabilitation of the cuff deficient shoulders. Performing exercises in scapular plane and higher abduction angles activates deltoid and other important muscles more efficiently and optimizes surgical outcomes.

## Introduction

The first shoulder replacement was performed for tuberculous arthritis by a French orthopedic surgeon, Jules E. Pean in 1893.^1^ The implant consisted of a platinum tube, a rubber ball coated with paraffin, and two metal loops that attached the ball to the scapula and the tube. The first generation of reverse total shoulder arthroplasty (RTSA) was introduced by German and French Surgeons in early 1970s but was discontinued quickly because of loosening, mechanical complications and inability to counter the superiorly directed force of the deltoid muscle in the absence of rotator cuff.^2^ A more efficient version of the RTSA was introduced by Paul Grammont, a French orthopedic surgeon^2–4^ in late 1970s. Grammont understood the importance of the balance between the supraspinatus-deltoid couple force and the role of the prosthesis’s centre of rotation (COR) in the cuff-deficient joint. Grammont felt that by medializing the COR of the glenohumeral joint and increasing the deltoid lever arm one could compensate for the lack of activity of the rotator cuff muscles, as cited by Baulot who worked closely with him.^2^ Grammont’s first modern prototype was manufactured in 1985 and was composed of an alumina ceramic sphered glenoid component with a medialized COR and a concave polyethylene cone cemented on the humeral site.^2^ The use of RTSA was approved in the United States by the Food and Drug Administration (FDA) in November 2003 and over the past two decades its indications which were initially limited to cuff tear arthropathy (CTA) have increased to include massive irreparable rotator cuff tears in the absence of osteoarthritis, proximal humerus fractures, glenohumeral osteoarthritis with excessive posterior glenoid erosion and revisions for failed anatomical arthroplasty.^5–11^

The literature on rehabilitation of the RTSA has been growing.^12–18^ Present guidelines are mostly based on the expert opinions and do not always address the influence of relevant muscle loading, altered joint biomechanics or unique complications of the RTSA.

The indications for RTSA have been increasing,^19–24^ but the post-surgical rehabilitation of cuff-deficient shoulders remains more challenging than those with a functioning rotator cuff.^25^ Therefore, further review of history, complications and muscle function will add to the body of knowledge in this area. The purpose of this article is to propose a rehabilitation protocol for a cuff-deficient glenohumeral joint following a RTSA by incorporating the principles of pertinent muscle loading and joint biomechanics while considering the potential for post-operative complications.

### Impact of Altered Biomechanics on Muscle Recruitment

In the rotator cuff-deficient glenohumeral joint, the humeral head is migrated superiorly with respect to the glenoid fossa due to lack of compressive forces of rotator cuff muscles.^26–28^ Traditional anatomic total shoulder arthroplasty is not a viable option for a rotator cuff deficient shoulder due to accelerated glenoid loosening caused by eccentric joint loading, excessive shearing forces and superior tipping of the glenoid component, a phenomenon referred to as the “rocking horse glenoid.”^29^ To reduce the risk of glenoid failure in a cuff-deficient shoulder, the semi-constrained design of modern RTSA prosthesis is comprised of a glenoid hemisphere with no neck and a humeral cup with a non-anatomical valgus angle. By distalizing the humeral component in relation to acromion, the deltoid muscle fibers are tensioned and recruited to elevate the arm while compensating for the deficiency of the cuff muscles.^8,30–33^

## Complications Following RTSA

Despite advantages of the RTSA, the reverse ball and socket model is associated with specific complications such as dislocation, scapular notching, acromion stress fracture and nerve palsy.^8,34–38^

### Dislocation

**Figure 1.**
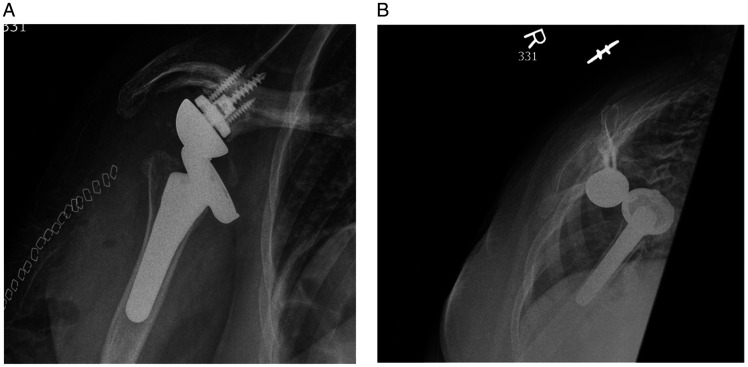
A, AP view of the right shoulder RTSA with humeral component displaced anteroinferiorly in a 53 year old woman. B, Transcapular view of the same patient showing anteroinferior dislocation of the humeral component.

The majority of post-operative dislocations after RTSA are anterior (Figure 1) and generally present within the first 5–12 weeks postoperatively. The most common risk factors for early dislocations are inadequate soft tissue tensioning, BMI >30, male gender, previous shoulder surgery and subscapularis deficiency.^39–41^ Late dislocations are mostly attributed to asymmetric wear of the polyethylene, male gender, rapid weight loss leading to excessive soft tissue with resultant lengthening or infection.^37,42,43^

In addition to the above risk factors, due to the unique semi constrained design of the RTSA, certain movements such as hyperextension, reaching across chest/abdomen or combined movements such as reaching behind back (adduction, internal rotation, extension) could increase the risk of anterior dislocation in the reverse model.^44^

### Scapular Notching

**Figure 2.**
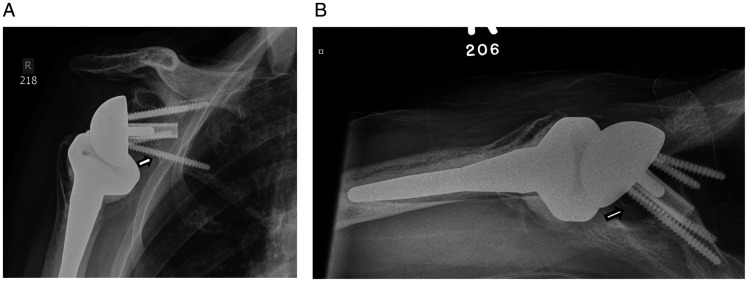
A, AP view of the prosthesis showing an erosive lesion at the inferior scapular neck in a 91-year female at 2 and ½ years post reverse arthroplasty. B, Axillary view of the same shoulder showing a linear lucency anteriorly along the component-cement interface at 2 and ½ years post reverse arthroplasty.

Scapular notching is a radiographic abnormality that refers to an erosive lesion of the inferior scapular neck secondary to impingement of the humeral implant during adduction,^45^ combined movements of flexion/extension and internal/external rotation with the arm at the side^46^ or chronic foreign-body reaction in the joint capsule (Figure 2).^47^ Scapular notching is specific to RTSA and appears to be more prevalent in the non-dominant extremity and in patients with low body weight.^48^ Patients with a physically demanding life style are also more susceptible to this pathology.^49^ The non-demographic risk factors for scapular notching are decreased pre-operative acromiohumeral distance, increased fatty infiltration of the infraspinatus, diagnosis, and type of glenoid erosion.^49^ Levigne and colleagues reported that cuff tear arthropathy was associated with 71% scapular notching as compared with 47% in those with osteoarthritis and cuff deficiency. In their study, patients with superior glenoid erosion had an incidence of 83%, where inferior glenoid erosion was associated with 25% scapular notching.^49^ Scapular notching may lead to glenoid implant loosening,^45,49^ humeral radiolucent lines^48^ and deterioration of patient-oriented scores and functional outcomes.^45,48,49^ Role of comorbidities such as Parkinson disease in RTSA is worth nothing. The limited research in this area reports reduction of pain but inferior clinical function and a much higher complication rates such as glenoid notching in these patients.^50,51^

### Scapular Stress Fractures

**Figure 3.**
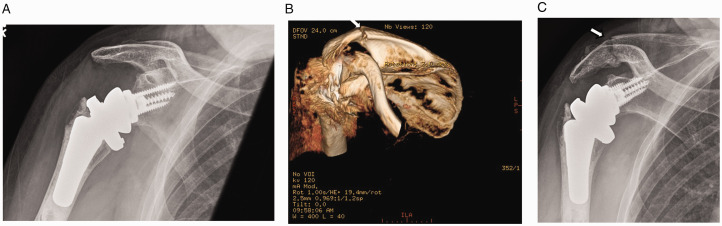
A, No obvious signs of stress fracture on plain radiographs in a 74 year-old male at 3 and ½ months post reverse arthroplasty. B, 1.2 mm helical CT scan images taken two weeks later showing healing fracture of the base of the acromion without significant displacement. C, Healing fracture of the base of the acromion now obvious on plain radiographs, taken two weeks after the CT scan and 4 weeks after the initial plain radiographs.

Increasing the deltoid moment arm is associated with improving the superior stability of the implant in RTSA. However, the longer arm length and greater deltoid tension increases the force on the origin of the deltoid muscle putting significant stress on the acromion (via middle deltoid) or the lower lip of the spine of the scapula (via posterior deltoid).^52,53^ Thus, stress fractures after RTSA can occur at various locations from the acromion to the scapular spine (Figure 3). The prevalence of stress fractures has been reported to vary from 0.6% to 15.8% according to a recent systematic review with a fairly similar rate of 50% for the acromial and scapular spine fractures.^54^ Factors associated with postoperative acromial stress fractures include osteoporosis, steroid use, prosthesis design, surgical approach and technical factors such as excessive lateralisation and humeral lengthening.^53,55,56^ The scapular spine stress fractures are less studied and are reported to be related to the cuff status, osteoporosis, glenoid wear, baseplate screw orientation, and implant design^56,57^ but the definite role of risk factors in their development remains unclear.^57^ Post-operative acromial and spinal stress fractures have a significant negative impact on pain and function with increased risk for revision surgery.^55–61^

### Neurological Deficits

The non-anatomic design of RTSA can lead to brachial plexus or axillary nerve injury (neuropraxia) mostly due to lengthening of the involved arm and elongation of the brachial plexus.^62^Van Hoof et al.^63^ reported a strain of up to 15.3% and 19.3% for the lateral and the medial root of the median nerve related to reverse shoulder prosthesis. Intraoperative factors such as arm manipulation, excessive traction or lengthening of the arm have been noted to contribute to nerve injury following RTSA.^64^ Fortunately, majority of neurological complaints are transient and show a full recovery over time.^62^

### Role of Pertinent Muscles in RTSA Rehabilitation

The non-anatomical design of RTSA alters the function of certain muscles which has implications for rehabilitation. Thus, to justify more specific strengthening exercises, we provide a short review of the anatomy and function of important muscles in the native shoulder and RTSA.

### Deltoid

In the native shoulder, the anterior deltoid has the largest abduction moment arm in flexion.^65^ The anterior deltoid also works with the subscapularis to internally rotate the humerus.^66^In RTSA, the anterior deltoid maintains its role in forward flexion and abduction.^67^ Ackland at al.^68^ reported that the anterior deltoid was involved in flexion but was a prominent contributor to abduction. According to Walker et al.,^69^ regardless of the type of implant used, anterior deltoid had the highest activation in abduction.

The middle deltoid is the main abductor with the supraspinatus initiating abduction.^70^In higher positions of elevation, the deltoid acts synergistically with the functioning rotator muscles (teres minor and infraspinatus if present). In this position, the deltoid has no upward component and participates more effectively in articular coaptation^71^ and flexion in the native shoulder.^65^ In RTSA, the middle deltoid is the most prominent shoulder abductor and in association with anterior deltoid and subscapularis produces a comprehensive force in the scapular plane.^68^ In the study by Li et al., the middle deltoid maintained its role more efficiently in abduction and to some degree in external rotation.^67^ Walker et al., recommended to strengthen the anterior and middle deltoid to synergize recruitment of the middle deltoid and trapezius at higher levels of elevation in cuff-deficient shoulders.^69^

The posterior deltoid is involved in extension and has a large external rotation moment arm, particularly during early abduction and flexion.^72^ The altered biomechanics of the RTSA increases the deltoid moment arm and recruitment of posterior deltoid as an abductor, but this comes at the expense of reduced axial rotation causing decreased external rotation commonly observed following RTSA.^73^ This phenomenon is more appreciable in patients with a tear or fatty infiltration of the infraspinatus as the infraspinatus and teres minor are the only muscles with appreciable moment arms for external rotation torque generation.^73,74^ While strengthening of the posterior deltoid may slightly improve external rotation,^67^a significant postoperative recovery of external rotation is generally not expected in patients with CTA unless infraspinatus and terse minor are intact.^69^ EMG studies have shown that even mild pre-operative fatty degeneration of teres minor can have a negative impact on gaining active external rotation. Patients with high grade fatty infiltration of teres minor might even experience a loss in external rotation after RTSA.^75^ The combination of latissimus dorsi transfer with RTSA has been reported to restore external rotation in patients with CTA and teres minor dysfunction.^76^

The importance of a healthy deltoid on active elevation and to a lesser degree on external rotation should not be underestimated in RTSA. Li et al showed a significant correlation between anterior and middle deltoid and postoperative flexion and abduction and between the posterior deltoid and external rotation.^67^ Greiner at al. established a relationship between pre-operative degeneration of the deltoid and shoulder weakness following RTSA.^77^ Yoon et al indicated that the pre-operative deltoid muscle volume significantly affected the post-operative functional outcome in patients with cuff tear arthropathy or irreparable cuff tears.^78^ The negative effect of fatty infiltration of the deltoid and infraspinatus has further been emphasized on post-operative subjective outcome scores and range of motion.^79^ Therefore, improving the efficiency of deltoid function both prior to and after surgery is a key factor in the rehabilitation of patients with cuff deficient shoulders.^18,31,32,68,73,80^

### Subscapularis Muscle

In the native shoulder, the subscapularis muscle functions predominantly as an internal rotator of the humeral head and is affected by the level of abduction of the shoulder joint.^81^ The lower subscapularis has been noted as a humeral head depressor and anterior stabilizer.^82^

The RTSA can be done through two approaches, the standard deltopectoral or the superolateral. The superolateral approach maintains the integrity of the subscapularis tendon and preserves the deltoid muscle which consequently minimizes postoperative immobilization and facilitates rehabilitation by allowing a more rapid active shoulder range of motion, without increasing the incidence of shoulder dislocation. Overall, the role of subscapularis muscle remains controversial in RTSA. While some studies support the subscapularis role in the prevention of prosthetic instability,^38,41,83–85^ others have stated that repair of the subscapularis does not affect the functional outcome after reverse total shoulder arthroplasty,^84,86^ particularly in a more lateralized design, raising concern over the potential impact on implant longevity due to its antagonistic effect on deltoid and external rotation.^87^ A recent systematic review has concluded that subscapularis repair after RTSA produces no clinically meaningful benefits, particularly using lateralized prosthetic designs.^88^Generally, should surgery include the release and repair of the subscapularis tendon from its insertion on the lesser tuberosity, the rehabilitation protocol should provide protection of the healing tendon.^89,90^For this reason, early passive external rotation that overstretches a healing tendon and active and resisted internal rotation exercises that strain the tendon should be limited.

### Scapular Stabilizers

The main stabilizers of scapula are the trapezius, serratus anterior, rhomboids, and levator scapula muscles and each play an important role in facilitating the optimal function of the shoulder. The upper fibers of the *trapezius* muscle are active during elevation of the native shoulder.^91^In arthroplasty patients, the higher pre-operative EMG activity of upper trapezius and deltoid was correlated with a better recovery of abduction, flexion and external rotation.^67^The authors proposed to rehabilitate the upper trapezius muscle in the middle range of abduction to help the middle deltoid muscle more efficiently.^67^

The *serratus anterior muscle* contributes to the upward rotation of the scapula during arm elevation and is maximally activated at shoulder flexion above 90°.^92–94^ The greatest activation for upper trapezius, serratus anterior and anterior and middle deltoid is reported to occur with external rotation at 90 of abduction.^95^

The *rhomboids* assist with scapular retraction. In normal shoulders, full retraction is essential in overhead activities and pulling motions.^96,97^Limitation in retraction can lead to increased stress on the anterior structures of the shoulder^97^ and cause anterior instability.^98^ Although, there is no specific study of rhomboid function in shoulder arthroplasty, strengthening of this muscle group would likely help with improving anterior stability.

The *levator scapulae* helps to elevate the scapula and tilt the glenoid cavity inferiorly by rotating the scapula downward. Research has shown that specific exercises to target this muscle are not necessary because strengthening of rotator cuff and other scapulothoracic musculature is an effective way of eliciting activity of the levator scapulae in TSA.^99^ The significance of altered biomechanics of the reverse glenohumeral joint on serratus anterior, rhomboids, and levator scapula has not been studied.

## Proposed Rehabilitation Following RTSA

In light of unique complications in RTSA and the altered role of muscles in a reverse arthroplasty, the rehabilitation should focus on strengthening of the relevant muscles while considering the potential post-surgical complications. At present, there are no guidelines with respect to consequences of exercise for scapular notching or stress fractures. Performing exercises in the scapular plane abduction with neutral humeral rotation helps to maintain optimal bony congruity between the humeral head and glenoid fossa as well as the optimal length-tension relationship of the scapulohumeral musculature.^100,101^ The rotator cuff muscles are more effective abductors in neutral and the deltoid is a more effective abductor at higher abduction angles.^102^ Therefore in CTA, where supraspinatus and infraspinatus are typically absent or dysfunctional, the abduction strengthening will be more beneficial with slight elevation in a more functional scapular plane. In addition, patient’s age, sex, BMI, comorbidity, bone stock and life-style/activity level have an impact on complication rate^37,52,53,56,60^ and need to be taken into consideration while prescribing an individualized management.

### Phase I: Early Post-op (Day 1–6 Weeks)

#### Precautions

The prohibited movements that remain in the precaution list for Phase I are internal rotation, adduction and extension either in isolation or combined. The activities that should be limited are tucking in a shirt, reaching behind back, reaching across the abdomen and chest, and moving the arm backwards. Lifting greater than 0.5 kg (weight of a coffee cup) and supporting the body weight using the surgical arm should be limited during this period. To avoid straining the structures beyond their integrity, some protocols have proposed lifelong precautions for lifting of more than 15 lbs,^15,18^ however, the exact amount of this limitation is not clear at this time.

During phase I which is usually about 4–6 weeks, the shoulder is immobilized in an abduction sling. The hand/wrist/elbow exercises and passive or active assisted range of motion of the shoulder are encouraged to avoid stiffness. During phase I, gentle pendulum exercises, periscapular exercises, passive or active assisted flexion limited to 90° (in lying position) are performed. Active assisted exercises will continue with the goal of increasing flexion to 120° by the 16^th^ week. Patients with good body mechanics may add flexion against a wall while using a towel or a ball to activate the serratus anterior muscle by pressing against the ball during the elevation.

### Phase II (6–12 Weeks)

#### Precautions

The clinicians should continue to enforce precautions for dislocation, particularly in active males and those with a high BMI. Despite lack of guidelines with respect to consequences of exercise for scapular notching, extensive painful adduction exercises are not recommended especially in medialized implants.^16,45^ The combined internal rotation, adduction and extension should be avoided for another 6 weeks. Performing repeated active flexion/extension and internal/external rotation at 0° of abduction could cause scapular notching and impingement that can lead to polyethylene debris and osteolytic reaction and have to be limited as well.^46^ Of interest, the glenoid impingement does not appear to occur for the internal/external rotation at 90° of abduction.^46^ Lack of glenoid impingement at higher abduction angles, may be an option for younger workers who have to perform repeated rotations as a part of their occupation (e.g. store clerk at the checkout line).

In terms of scapular stress fractures, too much stress applied through deltoid strengthening exercises, particularly in osteoporotic patients (e.g. disuse or steroid use)^52,53,56,60^ or in those with a low BMI^37^ is to be avoided during this phase of rehabilitation. Any sudden new symptoms associated with a declined active range of motion, localized tenderness, and pain on resisted deltoid activation may indicate a stress acromial or scapular spine fracture which should be followed up with CT scan^52,53,60^ to assess the amount of displacement of the acromion or spine of the scapula. In these cases, active and isometric exercises should be halted for 6–8 weeks or until union is confirmed on re-imaging. An abduction sling to decrease the deltoid tension is helpful in pain management and potentially reducing further displacement of the fracture.

**Figure 4.**
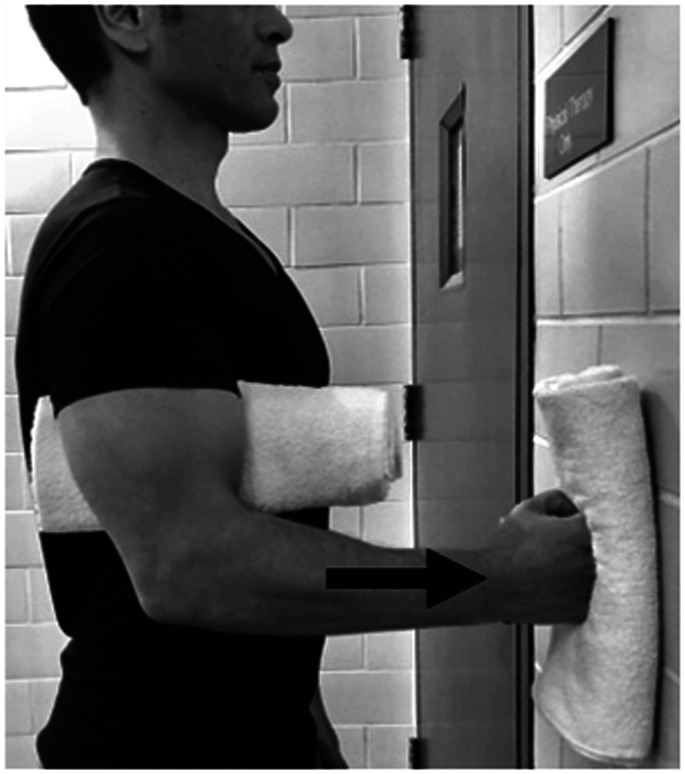
Anterior deltoid, pectoralis major and coracobrachialis. Isometric flexionin neutral position.

**Figure 5.**
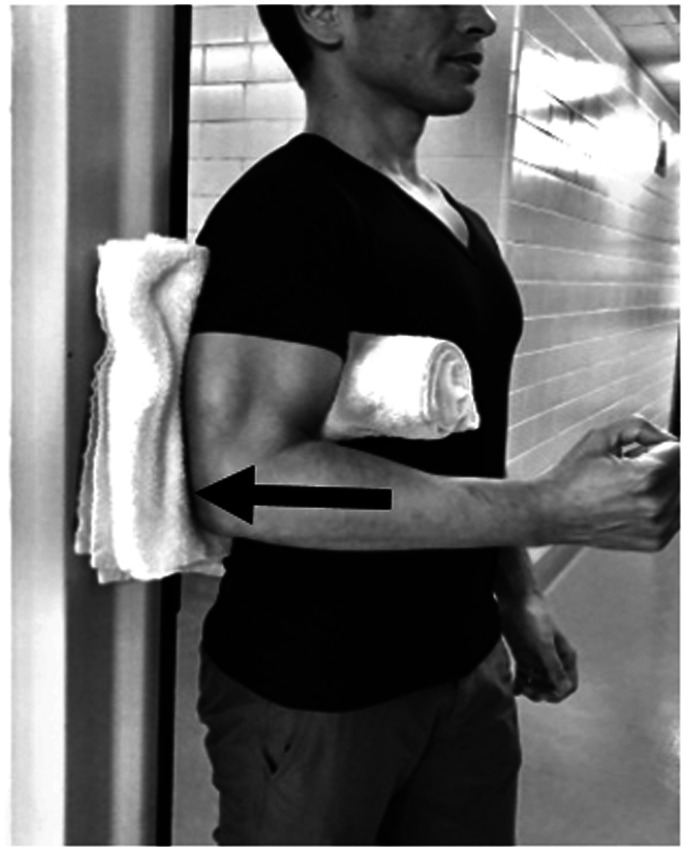
Posterior deltoid and latissimus dorsi. Isometric extension in neutral position. Hyperextension to be avoided at all times.

Active range of motion exercises and painfree submaximal isometrics in neutral position are initiated at this point. Isometric flexion and extension in neutral are the most basic exercises to activate anterior and post deltoid. Shoulder hyperextension while performing posterior deltoid strengthening should be avoided to minimize the risk of dislocation (Figures 4 and 5).

**Figure 6.**
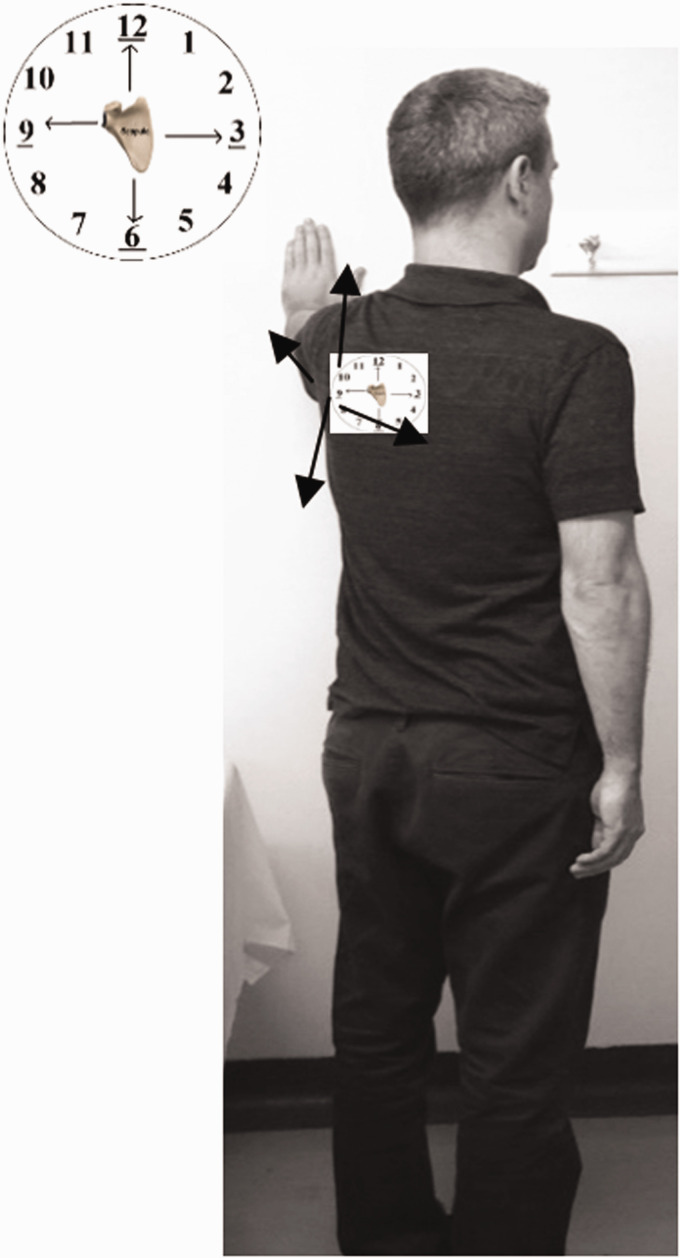
Scapular exercises (upper, middle and lower trapezius muscles, levator scapulae, rhomboids, latissimus dorsi and serrates anterior). • Face-clock exercises are done in standing, facing the wall with the hand pointed straight up and at any height that is comfortable. • Without moving hand during the exercise, the scapula is moved towards the numbers on the clock, starting with 12:00, 3:00, 6:00 and back over to 9:00. This will isolate the scapular stabilizers.

**Figure 7.**
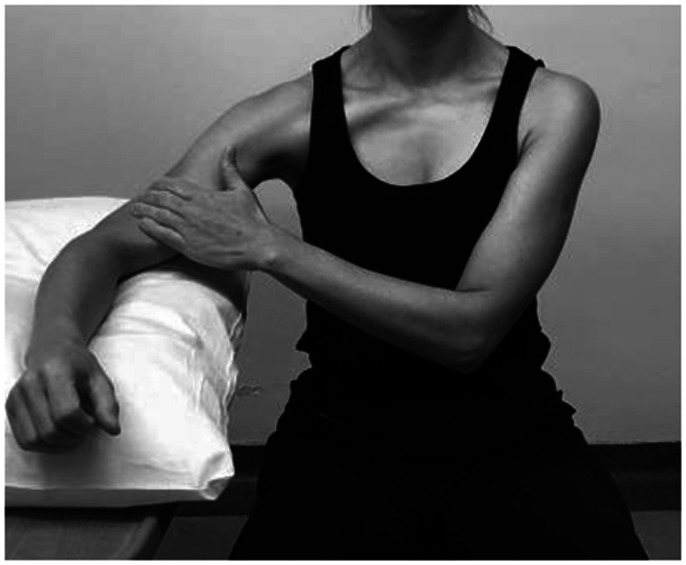
Anterior and middle deltoid. Isometric diagonal flexion and abduction in scapular plane.

**Figure 8.**
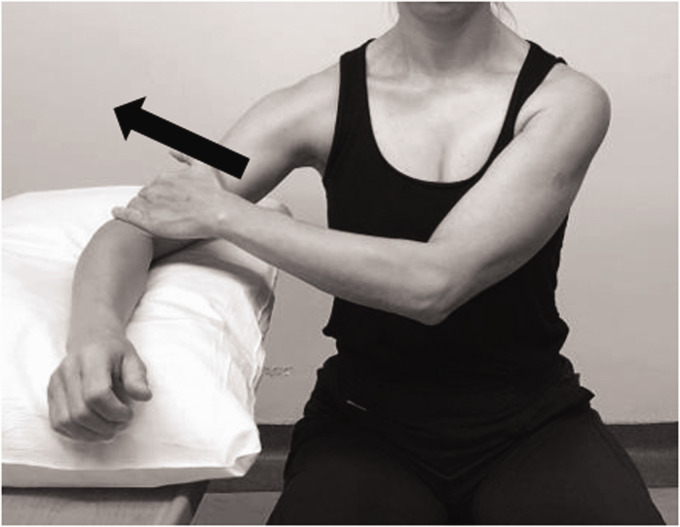
Middle deltoid. Isometric abduction in scapular plane to activate middle deltoid.

**Figure 9.**
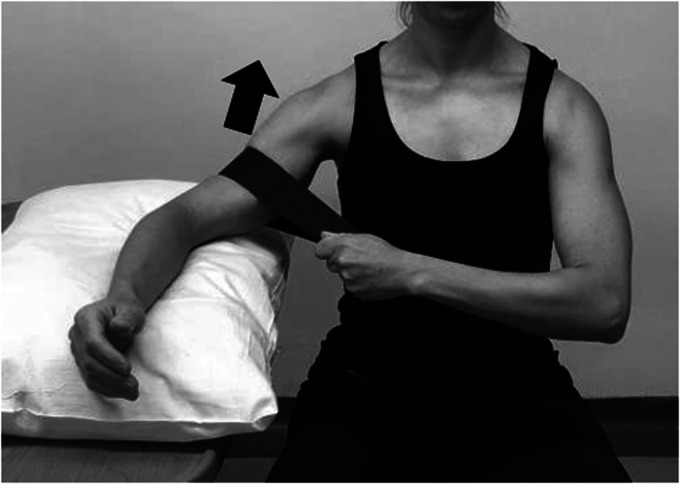
Posterior deltoid. Isometric diagonal extension and abduction in scapular plane to activate posterior deltoid. A strap is held by the opposite hand to provide resistance.

**Figure 10.**
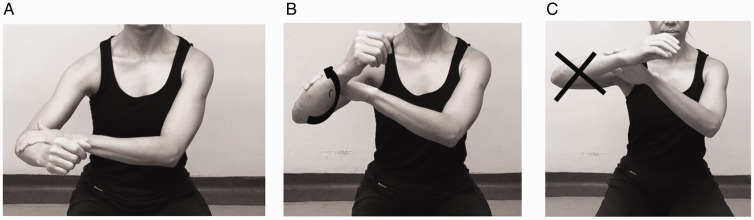
Posterior deltoid and teres minor. Isometric abduction and external rotation in the scapular plane. A, Initial position: affected arm is kept at 30° of scapular plane abduction and the forearm is pressed against the opposite hand outward. B, Progression: affected arm is externally rotated while resisting against the opposite hand and keeping the elbow inward. C, The hornblower sign, commonly seen in patients with CTA should be avoided by focusing on external rotation and moving the elbow inward.

The face-clock exercise will help to strengthen the scapular stabilizers (Figure 6). In a cuff-deficient shoulder, the scapular plane abduction activates different components of the deltoid as suggested by the literature.^68,69,71^ Isometric strengthening could therefore be facilitated by elevating the arm to about 30° of the scapular plane while resting on a table and isolating the anterior deltoid (Figure 7: diagonal flexion and abduction) from middle deltoid (Figure 8: predominantly abduction) and posterior deltoid (Figure 9: abduction/extension and Figure 10: abduction/external rotation).Excessive abduction of the arm during elevation is common in cuff deficient joints and is to be avoided by pulling the elbows inward while performing external rotation (Figure 10C).

In patients with a repaired subscapularis, submaximal isometric internal rotation may be started within 6–8 weeks according to surgeon’s preference to allow healing of the repaired tendon.^89,90^ If the subscapularis tendon was completely torn prior to surgery or was not repaired after surgery, this precaution does not apply. In the presence of subscapularis deficiency, teres major, latissimus dorsi and pectoralis major would play an important role in maintaining internal rotation.

### Phase III (>12 Weeks)

#### Precautions

Considering the possibility of a traumatic dislocation even after 12 weeks, the static and dynamic combined movements remain limited in high risk patients. Sudden and repetitive lifting, jerking activities, throwing weights, jumping, extensive hammering, punching (ballistic exercises) should be avoided^18^ as they may cause subluxation or dislocation of the implant.

Isotonic exercises using rubber-based resistance bands may be commenced for younger patients or those with a more active life style at this point. When using resistance bands, one should consider the length-tension relationship, which describes how much tension (i.e. load) is provided when the band(s) is stretched to a particular length. McMaster et al.^103^ provide a table that represents of the amount of weight added when the bands are stretched to a particular length. For example, the first 10 cm of the yellow resistance band adds about 2.8 kg, the first 20 cm adds about 5.7 kg. This load for the first 10 and 20 cm of the red resistance band is 4.6 and 9.6 kg respectively. Therefore, it is important for the clinicians to be aware of the strength and length of band used in this population.

**Figure 11.**
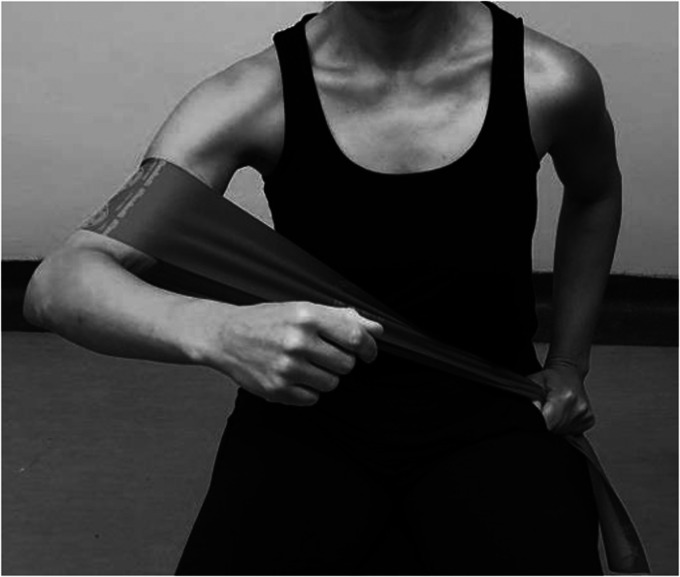
Anterior deltoid. Isotonic diagonal flexion and abdduction against resistance band in the scapular plane.

**Figure 12.**
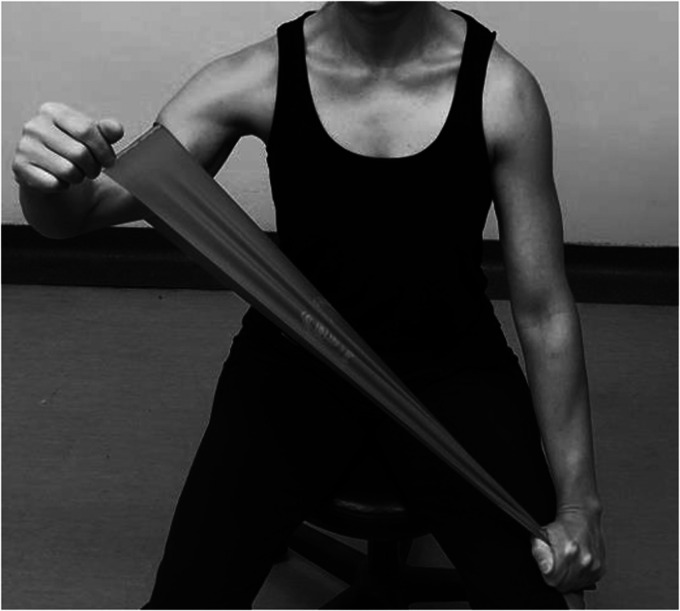
Middle deltoid. Isotonic abduction against resistance band in the scapular plane.

**Figure 13.**
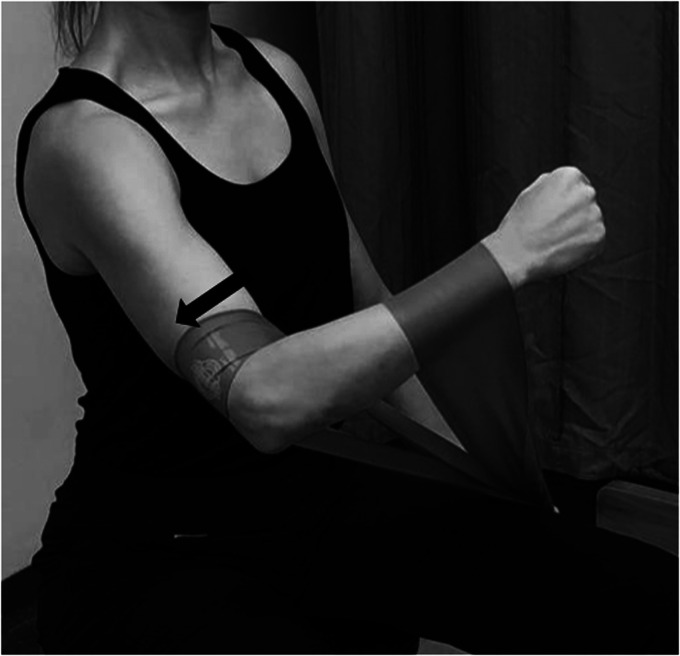
Posterior deltoid and teres minor. Advanced isotonic abduction and external rotation against resistance band in the scapular plane. While above elbow strap provides resistance in isometric abduction, the affected forearm is externally rotated against the resistance band above the wrist. Caution: limit the amount of stretch when using two bands simultaneously to avoid strain.

Most patients would have accomplished a painfree active range of motion and good deltoid activation and scapulothoracic rhythm. The stabilizing role of deltoid is more effective when the arm is elevated.^71^ Therefore, to better activate anterior deltoid, patient resists against a resistance band placed above the operated side elbow and held by the opposite hand at the hip level moving in a diagonal flexion and abduction direction (Figure 11). Middle deltoid isotonic strengthening involves resisting against a resistance band held by the opposite hand at the thigh level moving in the abduction direction (Figure 12). Forceful resistance band extension/abduction may not be safe for RTSA and in most cases an isometric posterior/extension as shown in Figures 4 and 5 is sufficient. The external rotation component of the posterior deltoid and the teres minor can be strengthened by placing a resistance band above the elbow and placing the second resistance band above the wrist, both held by the opposite hand. While the patient resists against abduction, the forearm externally rotates (Figure 13).

**Figure 14.**
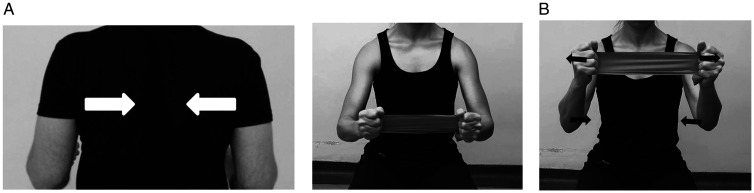
Ttrapezius, rhomboids, teres minor, anterior, middle and posterior deltoid. Bilateral isotonic arm elevation with external rotation using a resistance band. Elbows should be kept inward during the elevation. A, Initial position with squeezing shoulder blades. B, Final position.

**Figure 15.**
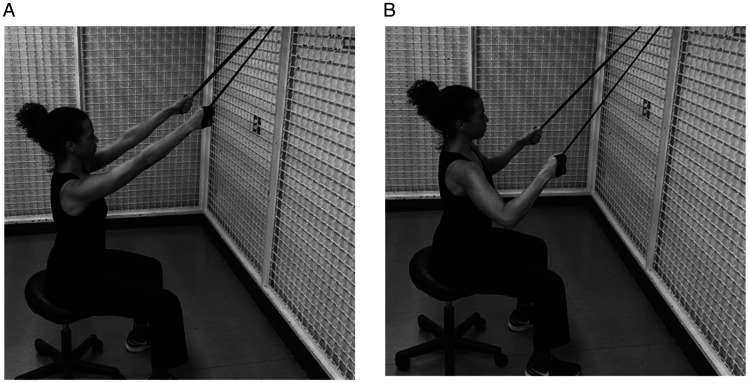
Posterior deltoid and latissimus dorsi. Isotonic lats/pull downs is performed with a band placed over top of a door or secured on a wall and the resistance band held in both hands. Patient pulls down in scapular plane abduction with the elbows in a 90° angle. Hyperextension to be avoided at all times. A, Initial position. B, Final position.

**Figure 16.**
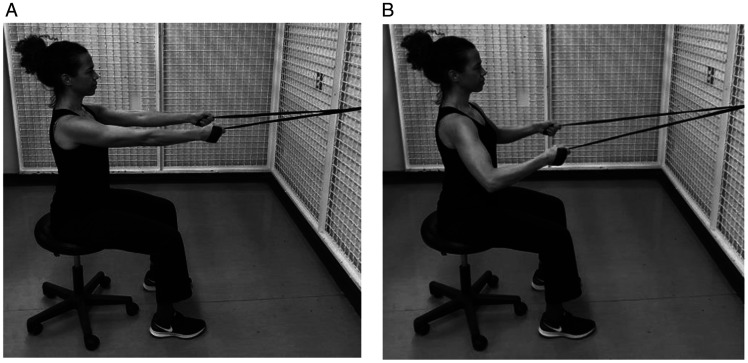
Posterior deltoid, rhomboids and latissimus dorsi. Isotonic rowing is performed with a band placed around a door knob or secured in the front. The resistance band is held in both hands and pulled backward maintaining the scapular plane abduction with the elbows in a 90° angle. Hyperextension to be avoided at all times. A, Initial position. B, Final position.

Performing bilateral symmetrical arm movements could help to improve proprioception via inter-hemispherical cerebral communication.^71^ The bilateral resistance band muscle strengthening is initiated at 30° of scapular plane abduction progressing to 90° while making the best effort to avoid the hornblower position commonly seen in this population (outward movement of the elbow) (Figure 14). Adding lats/pull down (Figure 15) and rowing (Figure 16) exercises would strengthen the posterior deltoid, latissimus dorsi, rhomboids and the overall trunk muscles. Patients are reminded to engage scapular muscles by squeezing their shoulder blades together during these exercises. It is suggested that if tolerated, these exercises be performed in standing with squats to help with core strengthening. Forceful shoulder hyperextension should be limited to neutral position at all times to avoid the risk of dislocation. A systematic review of rehabilitation protocols^17^ indicates that achieving 120°of active elevation is considered satisfactory as full active range of motion is not expected after reverse total shoulder arthroplasty. Following RTSA, potential for gaining significant external rotation remains small, particularly in the presence of fatty infiltration in teres minor.^75^

### Limitations

Given the lack of prospective comparative studies, the evidence to recommend for or against a specific timing of immobilization and initiation of passive, active and strengthening exercises is insufficient. Similarly, conducting randomized controlled studies to evaluate the impact of postoperative immobilization or certain exercises following RTSA for occurrence of dislocation, scapular notching or proximal humeral fracture may not be feasible due to the rare frequency of these complications and variability of the rehabilitation protocols. In this review, we have provided an assessment of the relevant muscles in RTSA and provided recommendations based on how the muscle function may be enhanced after this surgery. While this information is helpful for clinicians, future prospective or RCT may be needed to better assess different components of the rehabilitation in this population.
